# Retraining Dorsal Visual Pathways Improves Cognitive Skills After a Mild Traumatic Brain Injury

**DOI:** 10.3390/jcm14072273

**Published:** 2025-03-26

**Authors:** Teri Lawton, John Shelley-Tremblay, Roland R. Lee, Ming-Xiong Huang

**Affiliations:** 1 Cognitive Neuroscience Research and Remediation, Perception Dynamics Institute, Encinitas, CA 92023, USA; 2 Department of Psychology, University of South Alabama, UCOM 1123, Mobile, AL 36688, USA; jstremblay@southalabama.edu; 3 Department of Neurology, University of South Alabama, UCOM 1123, Mobile, AL 36688, USA; 4 Department of Radiology, VA San Diego Healthcare System, San Diego, CA 92161, USA; rrlee@health.ucsd.edu (R.R.L.); mxhuang@health.ucsd.edu (M.-X.H.); 5 Department of Radiology, University of California, San Diego, CA 92093, USA

**Keywords:** TBI rehabilitation, visual timing, improve cognitive skills, visual working memory, processing speed, attention

## Abstract

**Background and Objectives:** Currently, there are no proven solutions to remediate cognitive deficits in people with a mild traumatic brain injury (mTBI). One common issue is visual timing deficits, which may be due to processing deficits in dorsal visual pathways. **Methods**: This study investigates whether a new intervention (*PATH*) aimed at improving these visual timing deficits is more effective than conventional cognitive therapies that either remediate: (1) pattern discrimination deficits (ventral visual pathway): Orientation Discrimination (OD), or (2) working memory deficits using *ReCollect* task, for 10 subjects between the ages of 26–60 years old. This study tests the ability of three different cognitive therapies to improve the primary outcome: visual working memory (VWM), and secondary outcomes: processing speed, auditory working memory, and selective attention in mTBI subjects based on neuropsychological tests administered before and after 36 30-min training sessions Monday, Wednesday and Friday mornings. **Results:** On average, the *PATH* group exhibited a 35% improvement in VWM, compared to 15% for *ReCollect* and 5% for OD. A repeated-measures ANOVA found that improving dorsal stream function improved VWM significantly more than found after the other two interventions. The results reveal the importance of strengthening dorsal pathways more than conventional cognitive therapies to improve cognitive skills after mTBI. A biomarker, MagnetoEncephaloGraphy (MEG) brain recordings, using an N-Back task for subjects in treatment groups, verified these improvements as well. **Conclusions:** The data from this preliminary study are very promising for a new method improving the brain’s timing, more effective than conventional therapies, to improve cognitive deficits in mTBI patients.

## 1. Introduction

This study seeks to address the challenge of how to deliver targeted interventions that address the individualized cognitive needs of mild traumatic brain injury (mTBI) patients. mTBIs are prevalent, with over 2.5 million concussions occurring in the U.S. each year [1] with only 1 in 6 of these concussions being diagnosed. Concussions represent 80% of TBIs, with the bulk of concussions resulting from motor vehicle accidents, falls, and situations involving sudden acceleration and/or deceleration of the head, such as sports injuries [2]. Individuals suffering from a mTBI currently have limited treatment options. Currently, there are no proven solutions to remediate cognitive deficits prevalent in those with a mTBI [3,4,5]. Other cognitive training programs: (1) had little effect on improving the executive functions and attention in mTBI [6,7], (2) had results from brain training that were neither robust nor consistent, with transfer and sustained effects which were limited [8], and (3) improved the task being trained on, but do not extend to tasks not trained on or everyday cognitive performance [9]. None of these training programs address visual timing deficits that are prevalent in those with a mTBI. Substantial research [5,10,11,12,13] has found that the effects of a mTBI reflect disruptions of the neural networks for cognitive control. Working memory (WM), attention, and other executive function deficits are prominent cognitive sequelae of mTBI. WM deficits in mTBI do not improve over time [5]. Thus, daily living may be dramatically impaired after a mTBI. Visual timing deficits, resulting from magnocellular (motion-processing) deficits are persistent in individuals with a mTBI [14], manifesting as timing deficits in the dorsal pathways, and attention and executive control networks [14,15]. Many aspects of cognitive control deficits in those with a mTBI may in fact result from neural timing deficits [15]. Compensation for timing issues by increased prefrontal cortical recruitment would be manifest as increased distractibility, WM deficits, and problems with balance and coordination. This expended effort may underlie fatigue, headache, irritability, anxiety, and depression [16].

The scientific premise of this study is that remediation of a foundational visual timing deficit affecting motion discrimination at a low level of cognitive processing enables cognitive deficits at high levels of cognitive processing (working memory and attention) to be remediated. The literature on many species including humans has identified the specific cortical region vital for motion discrimination, widely known as middle temporal cortex (MT) [17]. Human MT (as determined by functional Magnetic Resonance Imaging (fMRI)) is located posterior to the Temporal-Parietal-Occipital junction in cerebral cortex [18,19]. Long white matter tracts connecting this region to pre-frontal cortex are especially vulnerable to damage from a mTBI [10]. The visual dorsal stream is composed of predominantly magnocellular (motion) neurons [20,21]. The dorsal stream provides information about ‘where’ an object is located, whereas the ventral stream provides information about ‘what’ high contrast, high resolution shape and color object attributes are present. The dorsal visual stream provides the input to the dorsal attention network [22], and WM networks [23]. We suggest that training low-level dorsal stream processes (movement discrimination) can be used to remediate mTBI related cognitive declines (WM, attention, and processing speed).

*PATH* neurotraining, a novel, patented [24] method, is designed to activate the dorsal stream at both: (1) low levels (visual motion areas), improving visual timing and movement discrimination, and (2) at high levels, improving attention, cognitive flexibility, processing speed, and working memory, improving high-level cognitive skills [15,24,25,26,27]. Therefore, *PATH* neurotraining improved cognitive abilities not related to movement discrimination, and improved everyday cognitive performance and quality of life in mTBI [15] and older adults [28], unlike other brain training programs that do not transfer to tasks not trained on. These improvements in visual timing enable the attention and executive control networks to function better. *PATH* training in mTBI patients [15] is the first time that improving low-level visual timing deficits in the dorsal stream were found to improve high-level cognitive functioning, both behaviorally and using a biomarker, MagnetoEncephaloGraphy (MEG) physiological brain recordings. *PATH* training had a much larger effect size for improving visual working memory [27] of d = 1.16 than found previously in a meta-analysis [29], d = 0.2, which included data from 115 studies and 2104 participants. Moreover, *PATH* training is more rapid, taking only 5–10 h, and more effective in improving cognitive skills, than competitive methods [25,26,27]. *PATH* training also increases in complexity, progressing from slower to faster movements, being an adaptive technology, which is essential for effective brain training [9,30]. Furthermore, evidence finds that improvements in cognitive skills after *PATH* training are sustained over time [25,31].

MEG recordings are established as a neural correlate biomarker of mTBI timing and cognitive functional improvements [13,32,33]. MEG functional brain imaging that measured responses evoked from an N-Back WM test on mTBI patients confirmed improvements in cognitive skills over their baseline in brain function after *PATH* training [15]. This pilot study illustrates the brain plasticity found in mTBI patients, that is, they show neurophysiological reorganization as evidenced by the MEG findings. The work of Hillary and colleagues [34] over the past decade has demonstrated that neural networks do change in their connectivity as a result of a mTBI. Our study is uniquely positioned to determine whether *PATH* can address the phenomenon of “hyperconnectivity” so often seen in mTBI patients. In response to neurological insult, the brain attempts to reorganize to increase information processing efficiency while bypassing damaged network connections. In the case where the damage occurs in a processing “hub” (e.g., perisylvian cortex damage yields Broca’s aphasia), this hyperconnectivity allows for as much compensatory information processing as possible but at the cost of local hypermetabolism. In diffuse mTBI the effect of prolonged hypermetabolism may actually be increased oxidative stress and long-term neural degeneration. By using MEG measures of functional connectivity, our team will see if the hyperconnectivity demonstrated in mTBI patients becomes ameliorated post treatment. Dr. Huang’s innovative methods for MEG source imaging [13,35,36,37,38,39,40,41] enable measuring timing changes, in terms of delta, theta, alpha, beta, and gamma oscillations, as well as changes in cognitive function following intervention training more precisely than any other methods. Statistical methods being used in this study to analyze MEG recordings and behavioral results utilize state-of-the-art techniques.

This study reports the preliminary results from a Phase I Small Business Innovation Research (SBIR) study to provide a proof-of-the-concept, within subject, investigation of the cognitive and behavioral effects of clinical trials for a 12-week treatment with *PATH* training compared to conventional methods to improve cognitive skills, in adults 18–60. Participants engaged in intervention training for 30 min 3 times/week for 12 weeks (36 training sessions). The premise of this preliminary study is rehabilitative treatments for those with a mTBI [3,4,5] fall short because visual timing issues are not being addressed. To address these limitations, a patented [24] movement-discrimination intervention (*PATH* training) that is designed to stimulate dorsal motion pathways [42,43] was developed. *PATH* has been shown to speed up the timing of visual events, increasing the sensitivity of both excitatory and inhibitory MEG signals in MT in the first 350 msec [31], and in turn improve cognitive functions that rely upon visual timing, such as working memory span [15,24,25,26,27], a function that is commonly impaired in mTBI patients [44]. Here we seek to test whether remediation of visual timing deficits, via *PATH* training, when followed by the digit memory task improves high level cognitive skills, i.e., visual working memory, more than conventional methods to improve these cognitive abilities. Support for this hypothesis can lead to better treatments for those with mTBI.

## 2. Materials and Methods

The study protocol was approved by Pearl IRB Institutional Review Board. All participants gave written informed consent prior to study procedures. The informed consent followed the ethical guidelines of the Declarations of Helsinki.

### 2.1. Human Subjects Involvement

This study recruited mild Traumatic Brain Injury (mTBI) patients between the ages of 18–60. These patients were referred by neurologists Dr. Alan Shahtaji at UCSD Concussion Clinic, Dr. Mohammed Ahmed at Kaizen Brain Center, a neuropsychologist, Dr. Shaul Saddick, a primary care physician, Dr. Maysa Nagi, the UCSD clinical trials website: https://clinicaltrials.ucsd.edu/trial/NCT03655782 (accessed on 16 July 2024), Heike Kessler-Heiberg, Associate Professor who taught Acquired Brain Injury class funded by State of California, and the San Diego Brain Injury Foundation (SDBIF).

In this preliminary study ten subjects were tested and trained at two coordinated sites: (1) UCSD MEG Center/Qualcomm Institute for MEG and MRI brain source imaging, and (2) Perception Dynamics Institute (PDI) for neuropsychological testing and intervention training which is located 10 min from UCSD. Training was performed at PDI or remotely on zoom. Since IRBs have determined that these cognitive interventions are of minimal risk and not yet proven to provide a proven treatment for mTBI, no FDA approval was needed before this study began.

Participants were randomly assigned to either the treatment: (1) Movement Discrimination training-*PATH* group or (2) the WM exercises-*ReCollect* group, or to the control (Orientation Discrimination) group as they entered the study, being distributed equally across the three groups. Pre-post standardized neuropsychological tests were administered to all subjects in this study. The mTBI subjects in the two treatment groups (*PATH*, *ReCollect*) had both standardized neuropsychological testing and MEG/MRI physiological recordings, providing a biomarker, a neural correlate, to determine whether the dorsal, attention, and executive control areas in the brain improve in function following *PATH* and *ReCollect* neurotraining. The three training groups were balanced in terms of age, sex, and scores on visual working memory, auditory working memory, processing speed, and selective attention, as verified by a one-factor ANOVA of pre-test standardized percentile scores. There is a fourth group: initially having a severe TBI, which is now a mild TBI, i.e., those who had a moderate TBI. Since this was a new group, all underwent *PATH* training and MEG/MRI imaging. None of these subjects had completed intervention training at the time we submitted this paper.

Subject confidentiality was protected by having a key connecting actual subject name and ID number in password protected data stored on a computer or kept in a locked file cabinet. Since data was collected automatically by all intervention training programs, and only the statistician, Professor Shelley-Tremblay: (1) entered behavioral data from subject’s pre- and post- tests into the REDCap database and FITBIR, and (2) analyzed the behavioral data, the PI, Dr. Lawton, did not influence the results.

Participants were two male subjects and 8 female subjects, between the ages of 26 to 60 years having a range of ethnicities that are typical for the San Diego area: 7 White (Non-Hispanic), 2 White (Hispanic), and 1 Asian (Non-Hispanic). Adults older than 60 were not included, since aging may be an additional factor in their cognitive decline. There were no exclusions on the basis of gender, race, or ethnicity, so we had a variety of ethnicities and races. The mTBI Human Subject Characteristics [16,45] and the Inclusion and Exclusion Criteria are described in the Supplementary Materials.

### 2.2. Human Subjects Design

**Figure 1:**
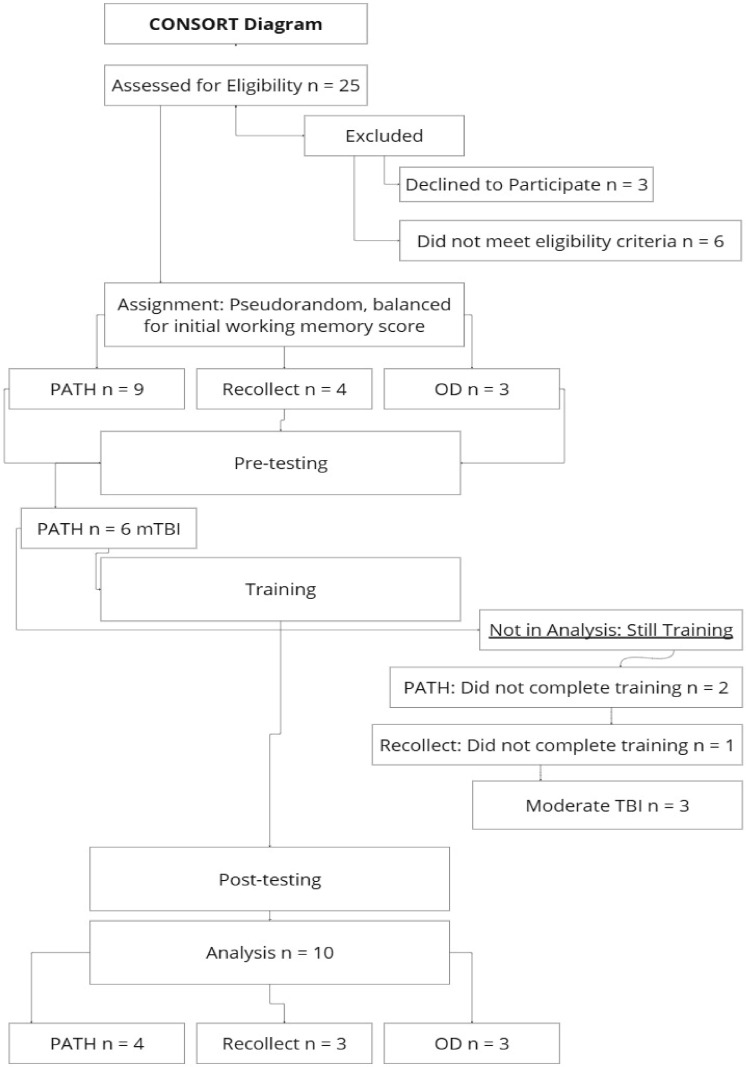
CONSORT Diagram for Study Enrollment, Randomization, and Analysis.

The CONSORT diagram in Figure 1 illustrates the flow of participants through the study, detailing eligibility assessment, randomization, training completion, and final analysis. A total of *n* = 25 participants were assessed for eligibility. Of these, *n* = 9 were excluded, with *n* = 3 declining to participate and *n* = 6 failing to meet eligibility criteria. The remaining *n* = 16 participants were assigned to one of four intervention groups using a pseudorandomized approach, ensuring balance for initial working memory scores: *PATH* (*n* = 6), *Recollect* (*n* = 4), and OD (*n* = 3), Moderate TBI *PATH* (*n* = 3).

Following pre-testing, *n* = 6 participants in the *PATH* group were identified as having a mild traumatic brain injury (mTBI). During the training phase, not all had completed training, with *n* = 2 from the *PATH* group, *n* = 1 from the *Recollect* group, and *n* = 3 from the moderate TBI group. Thus, *n* = 6 participants were still in training at the time of data analysis, and their data were not yet available.

Post-testing was conducted on *n* = 10 participants who completed the training phase. The final analysis included *n* = 4 in the *PATH* group, *n* = 3 in the *Recollect* group, and *n* = 3 in the OD group. This diagram provides a comprehensive breakdown of participant flow and attrition throughout the study, ensuring transparency in the reporting of the research process.

### 2.3. Assess Improvements in Cognitive Function in mTBI Subjects Aged 18 to 60 Years

Behavioral improvements in cognitive skills in this preliminary study were evaluated by comparing pre- and post- standardized percentiles on neuropsychological tests, see Supplementary Materials for the details of Behavioral Measures to Assess Improvements in Cognitive Skills (Pre-Post tests). The primary outcome measure: visual working memory was assessed using standardized percentiles on Test of Information Processing Skills (TIPS). The secondary outcome measures: auditory working memory (AWM) and processing speed were evaluated by administering the Wechsler Adult Intelligence Scale (WAIS-4) Working Memory Index (AWM), and Processing Speed Index, and Selective Attention by the Delis-Kaplan Executive Function System (DKEFS) Color-Word Interference test. Pre-post MEG imaging was used to determine whether there were improvements in dlPFC function, cortical area devoted to working memory, for subjects in the *PATH* group compared to subjects performing the *ReCollect* task.

### 2.4. Behavioral Measures to Assess Improvements in Quality of Life (Pre-Post Tests)

SF-36 The SF-36 Health Survey is a widely used measure of health-related quality of life, developed as part of the Medical Outcomes Study in the 1980s. It assesses eight domains of functioning, including physical functioning, bodily pain, and mental health, using 36 items. The SF-36 has demonstrated strong psychometric properties, with internal consistency reliability coefficients typically exceeding 0.80 for all subscales and test-retest reliability estimates ranging from 0.60 to 0.95, depending on the domain. Its construct validity has been well-established, with factor analyses supporting its two-component structure (physical and mental health). The SF-36 is commonly used in clinical trials, population health studies, and healthcare outcomes research due to its sensitivity to changes in health status.

### 2.5. Rivermead Post-Concussion Symptoms Questionnaire (RPQ)

The Rivermead Post-Concussion Symptoms Questionnaire (RPQ) was developed to assess the severity of post-concussion symptoms over time, particularly in mild traumatic brain injury populations. It consists of 16 items measuring physical, cognitive, and emotional symptoms, with respondents rating symptom severity on a 5-point Likert scale. Research has consistently shown the RPQ to have high internal consistency (Cronbach’s alpha > 0.80) and moderate to good test-retest reliability, particularly for persistent symptoms. Its validity is supported by correlations with other measures of concussion symptoms and health-related quality of life. The RPQ is commonly used in clinical and research settings to monitor symptom progression and evaluate treatment efficacy.

### 2.6. Test Whether PATH Training in mTBI Subjects Improves Working Memory More than Conventional Methods to Improve Working Memory

We determined whether *PATH* training improved visual working memory (VWM) more effectively than visual training using the same paradigm as *PATH* training except instead of using patterns stimulating dorsal stream function (low-contrast, low spatial-frequency, achromatic gratings), we used patterns that targeted ventral stream functions, (Orientation Discrimination) using high-contrast, colored and black/white sinewave gratings. Both of these 20-min visual training tasks were followed by a 10-min WM task, recalling from 5 up to 10 digits [46,47], increasing by one each time, since *PATH* is always followed by exercises to improve desired cognitive skills [48]. A second treatment group used *ReCollect* task, conventional training [49] that targeted dorsal lateral Prefrontal Cortex (dlPFC), a hub of the executive control network [23], in mTBI subjects. The *ReCollect* task is one of the most frequently used WM paradigms [50] to investigate the neural basis of WM processes. The choice of cognitive tests was informed by pilot data [15] suggesting that *PATH* training improves these cognitive skills (visual working memory) as well as processing in cortical visual motion areas, the hubs of the attention networks, and dlPFC, a hub of the executive control network [23].

*PATH to Reading/Insight^TM^* (*PATH*) training web-app (https://app.pathtoreading.com or https://pathtoreading.com, accessed on 16 July 2024), developed and manufactured by Perception Dynamics Institute, Encinitas, CA USA, is designed from the ground up to incorporate theory driven, empirically supported, approaches to vision training into an entertaining video game. Details of this game are described under Visual Timing Intervention. The cross-platform *PATH* training web-app: (1) incorporates approaches proven to boost perceptual learning, using patterns designed to stimulate magnocellular neurons (low-contrast achromatic sinewave gratings moving left or right) relative to a stationary background designed to entrain motion discrimination [42,43,51], (2) uses motivating tasks [48], and (3) coordinates attention and reinforcement [24,25,26,27] into a compelling video game, providing a framework where participants are confident in their performance [24,25,26,27] and receive consistent reinforcement to the training stimuli [48].

*PATH* training is a web-application that is implemented using modern architecture with Javascript as its core technology. This enables clients to use this app across all computers, iPads, and tablets on a Google Chrome browser. This web-app: (1) expands the mTBI customer/patient’s and therapist’s access to *PATH* training, (2) has dashboards showing graphical displays of data over time to improve feedback and supervision of patients, enhancing care provider access to monitor patient activity and assess progress, (3) improves user interface so all training steps are automated with either messages, FAQs, or short videos, ensuring therapy is used correctly, and does not require hands-on training, (4) builds an integrated PR system that reaches mTBI patients through the web, and social media, (5) has structured backend for easy data access by therapists, and (6) includes a HIPAA-encrypted backend that delivers *PATH* training using a secure and confidential worldwide database to improve visual and cognitive function, increasing market penetration using a low-cost, efficacious, disruptive, and transformative technology.

### 2.7. Study Procedures and Materials

This study determined whether training that targets visual timing improves cognitive skills and the function of brain pathways more rapidly and effectively than interventions differing in the targeted brain areas. Innovative methods to improve cognitive function in mTBI patients that differ from conventional approaches which focus on improving ventral pathways (pattern details) or frontal areas rather than dorsal pathways were investigated. This study determined whether an intervention that targets visual timing is more effective in improving cognitive skills than Orientation Discrimination (OD) or *ReCollect* training. A fundamental feature of the *PATH* intervention, unlike typical brain games, is that *PATH* trains perceptual skills using patterns that are specifically targeted to activate motion-sensitive (magno) cells relative to pattern-sensitive (parvo) cells in the early visual system. Our hypothesis is that through massed practice with these special patterns, using *PATH* to signal cells commonly associated with the dorsal pathway, the where system of the brain, and using OD to signal cells commonly associated with the ventral pathway, the what system of the brain, we can improve the brain function in these systems [25]. The *ReCollect* task targets working memory without improving low-level visual processing, as is true of *PATH* and OD training. A huge advantage of this type of behavioral training is that cognitive skills can be improved rapidly without the use of drugs or lengthy talk therapies. In addition to behavioral methods, magnetoencephalography (MEG) neuroimaging was used to test whether dorsal stream memory and attention networks improve in function significantly more following *PATH* neurotraining than following *Recollect* training.

#### 2.7.1. Visual Timing Intervention (PATH): Motion Direction-Discrimination Training

**Figure 2:**
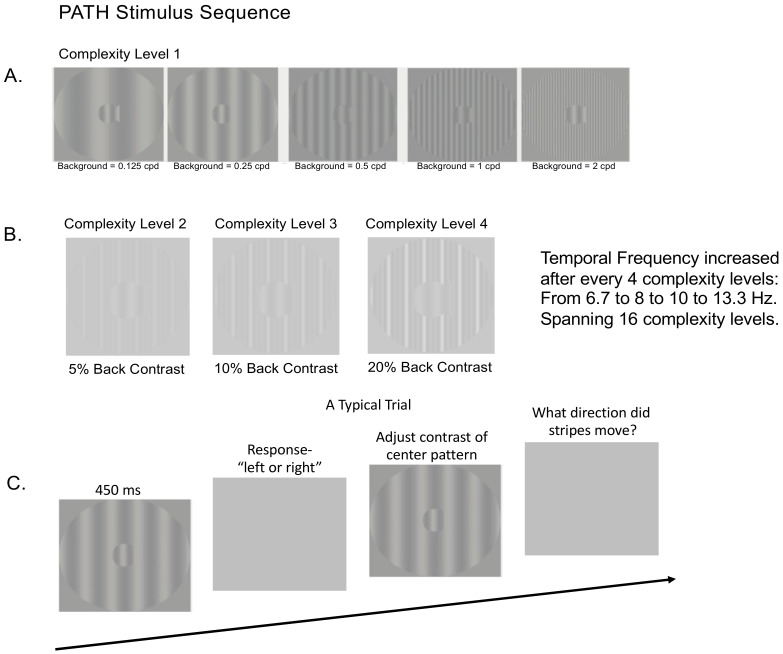
*PATH* Stimulus Sequence. (**A**) Sample patterns for *PATH* training at Complexity Level 1 for a background spanning ± two octaves in spatial frequency from the spatial frequency of the “fish shaped” test pattern (0.5 cyc/deg). This same set of backgrounds was presented in this order for each of the 4 test spatial frequencies (0.25, 0.5, 1, and 2 cyc/deg). (**B**) Complexity levels 2, 3, and 4 display multifrequency backgrounds for center pattern in 2.A, having the same fundamental frequency as in complexity level 1, with a difference frequency equal to the test frequency, increasing the background contrast from 5% to 10% to 20% contrast. (**C**) A typical trial for *PATH to Reading*/Insight intervention. Pattern flashes on the screen for ≤450 msec while the center stripes moved left or right. The screen goes blank, waiting for the left or right arrow key to be pushed. If incorrect, a short tone sounds. As soon as the left or right key was pressed, the next pattern with the same or different contrast flashes on the screen while the center stripes moved left or right. This sequence of patterns was presented continuously until the contrast threshold for this pattern was measured (20–40 trials). Then the next pattern combination was presented to measure the next contrast threshold until all 20 *PATH* neurotraining patterns were presented, and the program said ‘Thank You’, presented a star for each level of complexity completed, showed a graph with the contrast threshold function (optimal, current, initial) for each test frequency with its 5 background patterns, and quit.

The visual timing intervention, implemented using *PATH to Reading/Insight*^TM^ developed to remediate dorsal stream function, consists of motion direction-discrimination training. The mechanism that causes neural timing improvements is the repeated viewing of *PATH* stimuli that are moving left or right at increasing speeds relative to a stationary background, with sensitivity increasing the more *PATH* exercises are performed [25,48]. A sample of the stimulus patterns used in *PATH* neurotraining are shown in Figure 2 below. A more detailed description of the *PATH* neurotraining intervention is in the Supplementary Materials, and presented previously [31].

Once all 16 complexity levels of the Motion program were completed, the subject progressed onto the next program, the MotionMemory program which also had 16 complexity levels. MotionMemory required signaling the direction that two separate patterns move, one after the other, by pushing one of four arrow keys: the left arrow key if the stripes moved left and then right, the right arrow key if the stripes moved right and then left, the up arrow key if the stripes only moved right, and the down arrow key if the stripes only moved left. Thus, MotionMemory engages working memory more than required for the Motion program. The same motivational strategies used in the Motion program were used in the MotionMemory program.

For both Motion and MotionMemory programs: (1) Each contrast threshold in both programs required 20–40 trials to measure, using the most sensitive, repeatable measurements of contrast sensitivity possible [52]; (2) mTBI subjects usually completed a full *PATH* training cycle (one complexity level) on each visit, for 20 min (one training cycle) followed by the WM exercises (Digit Memory task) described below for 10 min for a total of 30 min; (3) Motivational strategies were presented at the end of each contrast threshold measurement: a fishnet appeared with a fish for each pattern having a contrast threshold ≤ 1% contrast, personal best score, current score, and number of patterns remaining; (4) The summary data could be accessed by clicking on the Performance tab on the dashboard; (5) Individual data was accessed by clicking on the Data tab on the dashboard; (6) By clicking on each contrast threshold in Data, a graph showing performance on each trial was shown, providing easy access to the subject’s performance; and (7) Data in the web-app are structured, with this study having a list of subjects, enabling easy access to each subject’s data.

#### 2.7.2. Control Intervention: Orientation Discrimination (OD) Training

**Figure 3:**
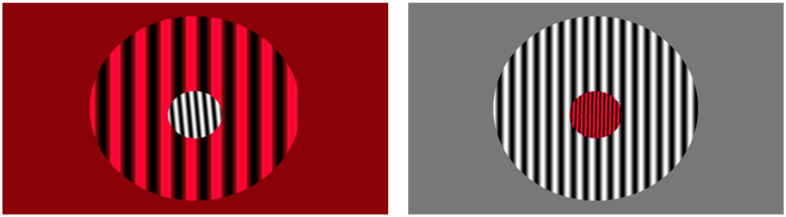
Sample patterns for Orientation Discrimination task.

The control intervention was Orientation Discrimination (OD) training that was identical to *PATH* training except instead of low contrast sine wave gratings moving left or right, high contrast (97%) stationary test and background sine wave gratings were used, both black and red gratings and black and white gratings; see patterns in Figure 3 below. This active control was critical because it controls for non-specific treatment effects not controlled for by a “no-treatment” condition. The benefits of this condition are also that it is more ethically sound than a true no-treatment control condition for individuals with *bona fide* deficits in need of intervention. These patterns that are randomly oriented left or right, were shown initially at a 0.1 radian tilt angle. As the test grating’s orientation was identified correctly, the grating’s orientation was presented at decreasing tilt angles. The subject pushed the left arrow key when the center pattern was tilted left and the right arrow key when the center pattern was tilted right. See Supplemental Materials for a more detailed description of the OD patterns. After the first incorrect response (signaled by a brief tone), the tilt angle was increased, using the same double staircase method used in *PATH* training to determine the orientation discrimination threshold (the smallest tilt angle able to be discriminated correctly from the vertical). mTBI subjects completed a full OD training cycle (one complexity level) on each visit for 20 min, followed by the WM exercises (Digit Memory task) described below for 10 min for a total of 30 min. Instead of activating dorsal pathways, the key element of PATH neurotraining, these patterns only activated parvo-cells in ventral pathways [53,54]. Therefore, the OD task does not speed up the brain’s visual timing, which is a function of the dorsal stream.

#### 2.7.3. Digit Memory Task to Improve Working Memory (WM) Skills

The subject followed the *PATH* and OD intervention training for 10 min with a Digit Memory task (*Retraining Dorsal Visual Pathways Improves Cognitive Skills*, *Patent Pending*) to exercise WM using a PowerPoint program. This program was used to provide the subject practice on working memory after *PATH* or OD intervention training. For the Digit Memory Task, a sequence of digits (large black numbers on a white background) were each presented for 500 msec in the center of the screen. The number of digits increased from 5 digits up to 10 digits, increasing by one digit each time, each sequence in different orders, with no repeating digits. Ten digits is near the limit of remembering in a 500 msec interval, since serial search (sequential processing) takes 20–45 msec per item [46], and previous items being presented must be kept in working memory. After the sequence was presented, a blank screen was shown, and then the subject wrote down the digits just seen and in the correct order. When the subject finished writing down all sequences from 5 up to 10 digits, they were given the answer key to correct mistakes made. Subjects practiced recalling the number of digits for up to four sequences of increasing digits, this task being limited to 10 min. Previous studies of *PATH* training found that practicing the cognitive skill that a subject wants to improve, e.g., reading speed, enables that cognitive skill to improve 11-fold instead of only 3-fold [25].

Sequential processing uses the functional anatomy of the claustral connections of items being processed serially, such that cross-frequency coupling between low frequency signals from the claustrum and higher frequency oscillations in the cortical areas is an efficient means of the claustrum modulating neural activity across multiple brain regions in synchrony [47]. Cross-frequency coupling is likely to play a crucial role in mediating working memory and in enabling learning, since it is being recognized as an efficient means of communication between different cortical areas [55,56]. Therefore, it is likely that following *PATH* training with the Digit Memory task increased coupled theta/gamma and alpha/gamma oscillations and visual working memory that are predicted to increase following *PATH* training. Including the Digit Memory task caused the visually based intervention training (*PATH* or OD) to take 30 min instead of 20 min to complete.

#### 2.7.4. Fidelity of Implementation

All training data with date and time stamps were stored in individual and summary files and collected automatically by software programs on each computer used to administer the *PATH* or OD training. Staff periodically examined the individual data from each subject to ensure they were on track. Since *PATH* is a web-app, all *PATH* data is now collected automatically in the Cloud, preventing any tampering with the data collection. The tasks were adaptive, increasing the complexity level so the task gradually increased in difficulty. Also, feedback was given during game-play and at the end of sessions to encourage participants to perform their best. Many motivational strategies, like catching fish in a net when the contrast for movement discrimination was 1% or less, one star for each level of complexity completed, graphs showing progress, were implemented to teach a subject to learn the task quickly. If the task was not being performed correctly, e.g., obtaining a high score, then the RA or PI helped the subject use better strategies to learn the task. It is essential for a subject to perform the task correctly to improve cognitive skills.

#### 2.7.5. ReCollect Task Intervention

A number of studies have examined how WM processes can be strengthened through training. The conventional approach is to target the central process component of WM models with the assumption that strengthening this process results in transfer across tasks involving WM [57]. A prototypical example is the “n-back” task; a WM updating task requiring participants to memorize and constantly update serial positions “n steps back” in a continuous stimulus stream. The task was to report when a current stimulus matched the stimulus n-items back. N-back training, in which task difficulty (level of n) was adaptively adjusted to participants’ performance, reportedly producing generalizing effects in healthy young populations [57], older adults [58], and typically developing children [59]. The training-related improvements were wide-ranging, from non-trained WM functions [60], to episodic memory [61], and even fluid reasoning [62]. Those behavioral changes were accompanied by changes in brain structure and function [63,64,65]. While the extent of transfer to cognitive domains after n-back training is still debated [66,67], several recent meta-analyses [68,69,70] show overall consistencies across studies suggesting that research should address the factors that mediate and moderate the magnitude and generality of learning rather than simply focusing on whether or not n-back training is efficacious.

Here we used *ReCollect the Study* (a cross-platform WM game developed by Dr. Aaron Seitz, Northeastern University). In this task, participants were required to compare each item to the item that they saw n-items back in the sequence; see ReCollect Task and Figure S1 in Supplemental Materials for a more detailed description of this task. The subject performed this task for 15 min, followed by a Span task: identifying the order of different colored flowers to be placed in a flower box, the number of colored flowers increasing as performance improved, for 15 min 3 times/week for a total of 30 min.

#### 2.7.6. Fidelity of Implementation

All training data with date and time stamps were stored in individual and summary files in the cloud, and collected automatically by software programs on each Android tablet used to administer the training, preventing any tampering with the data collection. The task was adaptive, which helped with compliance as when difficulty was too high then it automatically changed to become easier. Also, feedback was given during game-play and at the end of sessions to encourage participants to perform their best.

#### 2.7.7. Use MagnetoEncephaloGraphy (MEG) to Test Whether Dorsal Stream Memory and Attention Networks Improve in Function Significantly More Following PATH Neurotraining than Following Recollect Training

We employed MEG pre-post recordings to understand the distribution of timing-based deficits across 6 mTBI patients in the treatment groups, four in the *PATH* group and two in the *ReCollect* group, to evaluate whether they had significant improvements in the functioning of cortical networks after intervention training. MEG provides a unique biomarker of timing deficits in mTBI. Resting-state MEG is extremely sensitive in detecting neuronal abnormalities in a mTBI [32,35,36,37,38,39,40,41] on an individual-subject basis. Moreover, those who show large differences in MEG recordings should be those who show the largest behavioral improvements. Preliminary data suggest that MEG imaging is sensitive in detecting brain functional changes in the dorso-lateral Pre-Frontal Cortex (dlPFC), and anterior cingulate cortex (ACC) which are part of the VWM network. While these data support feasibility, a larger sample will be required to determine the reliability of these changes in dlPFC and ACC. The data from the Phase I study when it is completed will be used to get effect sizes on how *PATH* training impacts key mechanisms that are thought to mediate the behavioral changes, to guide the Phase II design.

Before initial MEG recording, a structural MRI used for superimposing the functional activity on top of the brain anatomy was performed, as is always performed in MEG studies to increase the spatial resolution of the analysis [71]. Dr. R.R. Lee, a board-certified neuroradiologist and diagnostic radiologist, consulted on MRI/MEG imaging to provide diagnosis of any cognitive abnormalities in mTBI Subjects. To examine the functional changes in the (1) visual system, the magnitude of improvements in MEG physiological recordings in the first 100–200 msec interval was used, and (2) changes in the later responses of the WM network, the 200–1000 msec interval were used to evaluate the effectiveness of *PATH* compared to *ReCollect* training. Physiological recordings, voxel-wise MEG source magnitude images, enabling 1 mm resolution across space and over time, were recorded by Professor Ming-Xiong Huang and his staff. To evaluate improvements in brain function MEG images covering the whole brain, each frequency band following the Fast-VESTAL procedure [32,33], to measure time-locked signals during WM task was used, as performed in a pilot study [15]. For each mTBI subject who qualified for imaging, two MEG exams were performed, one before and another after the brain training treatment.

Participants underwent MEG recordings while performing an N-back (N = 2) WM task. The task entailed on-line monitoring, updating, and manipulation of remembered information. During the task, the participant was required to monitor a series of letters (both upper and lower case) presented for 500 ms in the middle of the screen. A fixation cross was presented during the 3000 ms interstimulus interval. The participant was instructed to respond only when a letter was presented that matched (i.e., target) the one presented *n* trials previously, while not to respond to the unmatched stimuli (non-target). Two load conditions were used (1-back and 2-back), which placed increased demands on WM processes. About 50 trials per load condition were collected for each participant. Performance was recorded using a MEG-compatible response pad, in which index finger blocked-and-unblocked a laser-beam.

The MEG WM letter-based N-back task and the process of processing MEG responses were described in detail previously [13]. In the present study, ANOVA with repeated measures was used to assess the group differences (i.e., mTBI through *PATH* vs *ReCollect* treatment) for the treatment effect (i.e., post- vs pre-treatment differences). Family-wise error across voxels was corrected using standard cluster analysis for the ANOVA maps to control for family-wise errors at a corrected *p* < 0.01 level, using “3dFWHMx” and “3dClustSim” functions in the latest version of AFNI (http://afni.nimh.nih.gov). A mask that contained the statistically significant clusters was created, and then applied to the ANOVA maps to create the corrected group statistical maps for the MEG source magnitude images.

## 3. Results

### 3.1. Visual Working Memory (VWM)

**Figure 4:**
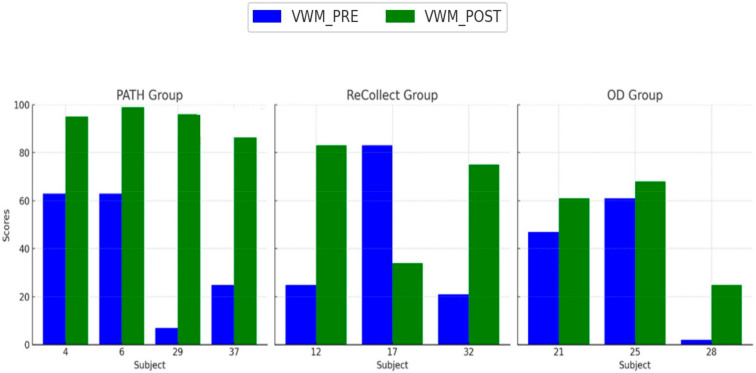
Visual Working Memory (VWM) PRE and POST scores for each subject across the *PATH*, *ReCollect*, and OD groups. Scores represent performance on the *Test of Information Processing Skills (TIPS)* before (VWM_PRE, blue) and after (VWM_POST, green) intervention. The *PATH* group exhibited the largest improvements in VWM scores, followed by the *ReCollect* group. Minimal improvement was observed in the OD group. Individual variability in response to training is evident, particularly within the *PATH* group.

The purpose of this analysis was to determine whether *PATH* training improves Visual Working Memory (VWM) as measured by the *Test of Information Processing Skills (TIPS)* compared to traditional methods (*ReCollect*) and the control group (OD); see Figure 4 below. Participants in the *PATH* group demonstrated an increase in VWM scores from PRE to POST testing, with the largest improvement in scores relative to the *ReCollect* and Orientation Discrimination (OD) groups. The *ReCollect* group exhibited smaller gains in VWM, and the OD group showed minimal or no improvement in VWM scores. On average, the *PATH* group exhibited a 35% improvement in VWM, compared to 15% for *ReCollect* and 5% for OD groups. To analyze differences, a repeated-measures ANOVA was conducted, with Group (*PATH*, *ReCollect*, OD) as a between-subjects factor and Time (PRE, POST) as a within-subjects factor. The repeated-measures ANOVA was selected due to its ability to detect interaction effects between Group and Time. A significant main effect of Time was observed (F(1, 9) = 12.54, *p* = 0.003), indicating overall improvement in VWM scores from PRE to POST testing. There was also a significant Group × Time interaction (F(2, 9) = 9.87, *p* = 0.002), suggesting that the *PATH* group improved significantly more than the *ReCollect* and OD groups. Post-hoc comparisons revealed that *PATH* training resulted in significantly greater improvements in VWM, particularly among subjects 29 and 37, than the other groups (*p* < 0.01).

### 3.2. Auditory Working Memory (AWM)

**Figure 5:**
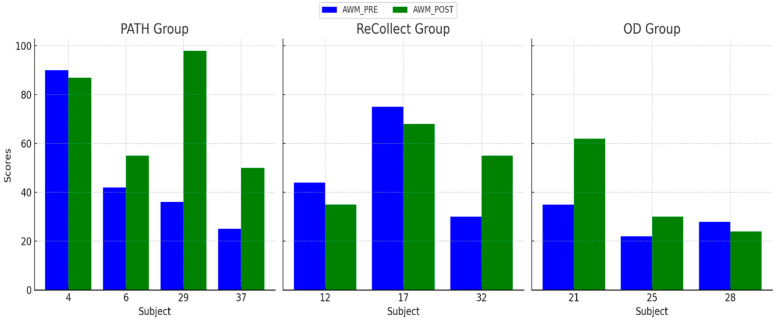
Auditory Working Memory (AWM) PRE and POST scores for each subject across the *PATH*, *ReCollect*, and OD groups. Scores represent performance on the *WAIS-4* Working Memory Index before (AWM_PRE, blue) and after (AWM_POST, green) intervention. The *PATH* group demonstrated the largest improvements in AWM scores, particularly among subjects 29 and 37. The *ReCollect* group showed moderate improvements, while the OD group exhibited minimal or no changes in AWM scores. Individual variability is evident across all groups.

The purpose of this analysis was to evaluate improvements in Auditory Working Memory (AWM), that were assessed by examining the *WAIS-4* Working Memory Index; see Figure 5 below. Participants in the *PATH* group showed modest improvements in AWM scores, with gains more pronounced in the Letter-Number Sequencing subtests compared to Digit Span subtests. The *ReCollect* group exhibited moderate improvements, while the OD group showed little to no change in AWM scores. A non-parametric Friedman test was conducted to assess differences across groups and time points due to the small sample size and deviations from normality observed in the AWM POST scores. Pairwise Wilcoxon signed-rank tests were used for post-hoc comparisons. The Friedman test indicated a significant difference in AWM improvements across groups (χ²(2) = 8.45, *p* = 0.015). Post-hoc tests revealed that the *PATH* group outperformed the OD group (*p* < 0.01) and the *ReCollect* group (*p* < 0.05) in AWM improvements.

### 3.3. Processing Speed Index (PSI)

**Figure 6:**
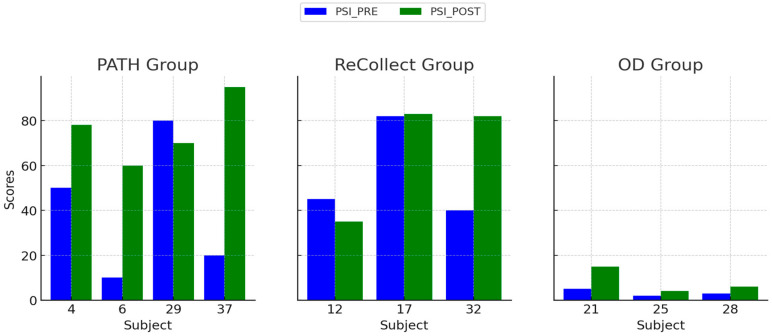
Processing Speed Index (PSI) PRE and POST scores for each subject across the *PATH*, *ReCollect*, and OD groups. Scores represent performance on the *WAIS-4* Processing Speed Index before (PSI_PRE, blue) and after (PSI_POST, green) intervention. The *PATH* group demonstrated the largest improvements in PSI scores, particularly among subjects 6 and 37. The *ReCollect* group exhibited moderate improvements, while the OD group showed minimal changes in PSI scores. Individual variability in response to training is observed within all groups.

This analysis aimed to assess the effects of different types of intervention training on Processing Speed, measured using the *WAIS-4* Processing Speed Index (PSI), is shown below in Figure 6. The *PATH* group exhibited significant improvements in PSI scores from PRE to POST testing, driven primarily by gains in the Digit Symbol Coding subtest. The *ReCollect* group showed moderate improvements, and the OD group displayed little to no change in PSI. A two-way mixed ANOVA was conducted, with Group as a between-subjects factor and Time as a within-subjects factor. Homogeneity of variances was verified using Levene’s test, and the assumption of sphericity was checked. A significant main effect of Time was observed (F(1, 9) = 10.34, *p* = 0.006), indicating improvements in PSI scores across all groups. The Group × Time interaction was also significant (F(2, 9) = 7.56, *p* = 0.009), with post-hoc tests showing that the *PATH* group improved significantly more than the OD group (*p* < 0.01).

### 3.4. Selective Attention

**Figure 7:**
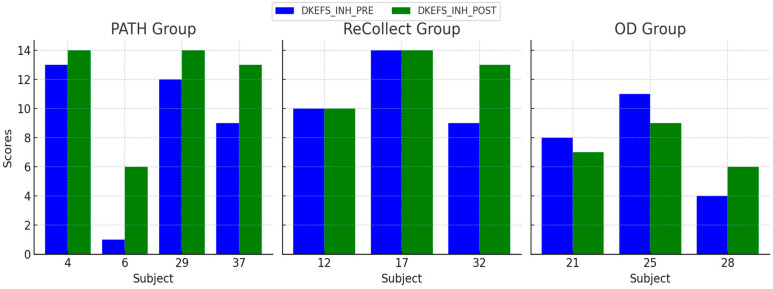
DKEFS Inhibition Scaled Scores (DKEFS_INH) PRE and POST for each subject across the *PATH*, *ReCollect*, and OD groups. Scores represent performance on the Delis-Kaplan Executive Function System (DKEFS) Inhibition test before (DKEFS_INH_PRE, blue) and after (DKEFS_INH_POST, green) intervention. The *PATH* group demonstrated substantial improvements, particularly among subjects 6 and 37. The *Recollect* group exhibited moderate gains, while the OD group showed minimal changes in DKEFS_INH scores. Individual variability is observed across all groups, with the *PATH* group showing the greatest overall improvements.

This analysis examined improvements in Selective Attention that were assessed using the Delis-Kaplan Executive Function System (DKEFS) Color-Word Interference Test (Stroop test); see Figure 7 below. The *PATH* group demonstrated substantial improvements in Stroop task performance, with reduced reaction times and errors. The *ReCollect* group showed moderate improvements, while the OD group displayed minimal changes. A Kruskal-Wallis test was conducted due to deviations from normality in the POST scores. Pairwise Mann-Whitney U tests were used for post-hoc comparisons. The Kruskal-Wallis test revealed significant differences between groups (H(2) = 11.56, *p* = 0.004). Pairwise comparisons indicated that the *PATH* group significantly outperformed both the OD (*p* < 0.01) and *Recollect* groups (*p* < 0.05).

### 3.5. Quality of Life

#### 3.5.1. Rivermead Post-Concussion Symptoms Questionnaire (RPQ)

**Figure 8:**
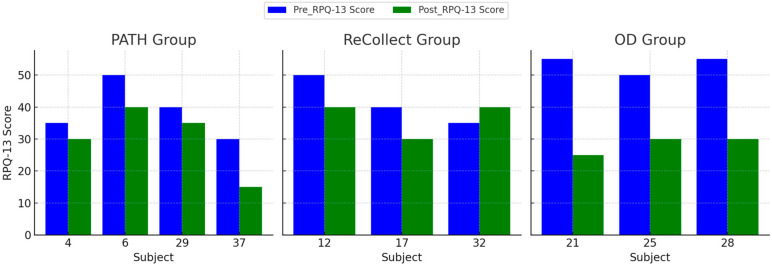
Mean RPQ-13 scores for the OD, *PATH*, and *ReCollect* groups at PRE-test and POST-test. The RPQ-13 score is a measure of post-concussion symptoms, with higher scores indicating greater symptom severity. All groups showed a decrease in RPQ-13 scores from PRE-test to POST-test, suggesting an overall reduction in symptom severity over time. However, the degree of change varied between groups, with the OD group showing the largest reduction, indicating potentially greater improvement in symptom management or recovery compared to the *PATH* and *ReCollect* groups.

The RPQ-13 score, see Figure 8 below, is a derived subscore of the Rivermead Post-Concussion Symptoms Questionnaire (RPQ) that focuses on cognitive and emotional symptoms rather than physical symptoms. A repeated measures ANOVA was conducted to examine the effects of Time (Pre vs. Post) and Group (*PATH*, *ReCollect*, OD) on RPQ-13 scores. The within-subjects effect of Time was significant, F(1,7) = 16.53, *p* = 0.005 F(1, 7) = 16.53, *p* = 0.005, indicating a substantial reduction in RPQ-13 scores from PRE to POST intervention. The interaction between Time and Group was not significant, F(2,7) = 1.34, *p* = 0.322 F(2, 7) = 1.34, *p* = 0.322, suggesting that the pattern of change over time did not significantly differ among the groups.

For the between-subjects effects, the main effect of Group was not significant, F(2,7) = 0.950, *p* = 0.432 F(2, 7) = 0.950, *p* = 0.432, indicating no overall differences in RPQ-13 scores between the *PATH*, *ReCollect*, and OD groups. Estimated marginal means suggest significant reductions in RPQ-13 scores across all groups over time, with consistent improvements irrespective of group membership. These findings highlight that the intervention was effective in reducing RPQ-13 scores, but the magnitude of improvement was similar across all groups.

#### 3.5.2. Quality of Life (SF-36)

**Figure 9:**
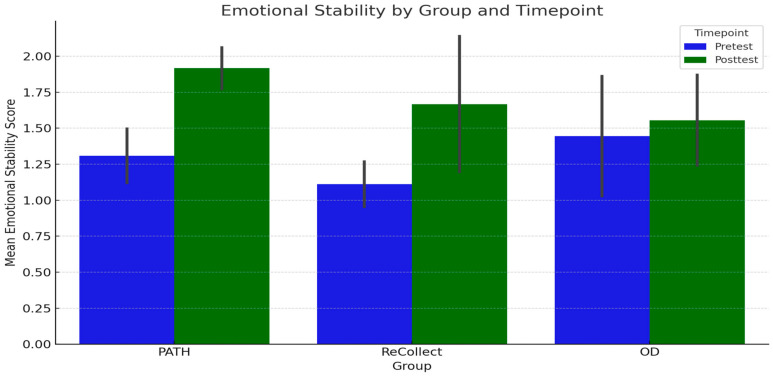
Mean Emotional Stability scores at PRE-test and POST-test for each group (*PATH, ReCollect*, and OD). Error bars represent standard deviations. Emotional Stability scores increased significantly from PRE-test to POST-test across all groups (*p* = 0.018), but no significant differences were found between groups (*p* = 0.469). This suggests consistent improvements in Emotional Stability scores over time, regardless of group membership.

Descriptive statistics for Emotional Stability scores indicate that the *PATH* group had a mean score of 1.31 (SD = 0.22) at PRE-test and 1.92 (SD = 0.17) at POST-test, reflecting a notable increase over time. The *ReCollect* group showed a similar pattern, with a mean score of 1.11 (SD = 0.19) at PRE-test and 1.67 (SD = 0.58) at POST-test. The OD group demonstrated a smaller increase, with a mean score of 1.44 (SD = 0.51) at PRE-test and 1.56 (SD = 0.38) at POST-test. A repeated-measures ANOVA was conducted to examine the effects of Timepoint (PRE-test vs. POST-test), Group (*PATH*, *ReCollect*, and OD), and their interaction on Emotional Stability scores. The main effect of Timepoint was significant, F(1, 42) = 14.35, *p* = 0.001, indicating that Emotional Stability scores increased significantly from PRE-test to POST-test across all groups. The main effect of Group was not significant, F(2, 42) = 1.24, *p* = 0.30, suggesting no differences in Emotional Stability scores between the *PATH*, *ReCollect*, and OD groups. To further explore the effect of Timepoint within the *PATH* group, a simple main effects analysis was conducted. The results revealed a significant simple main effect of Timepoint for the *PATH* group, F(1, 6) = 19.81, *p* = 0.004, demonstrating that Emotional Stability scores significantly increased from PRE-test (M = 1.31, SD = 0.22) to POST-test (M = 1.92, SD = 0.17). These findings suggest that Emotional Stability improved over time regardless of group membership, with significant within-group improvement observed for the *PATH* group. See Figure 9 for a visual representation of these results.

### 3.6. Neuroimaging Results

**Figure 10:**
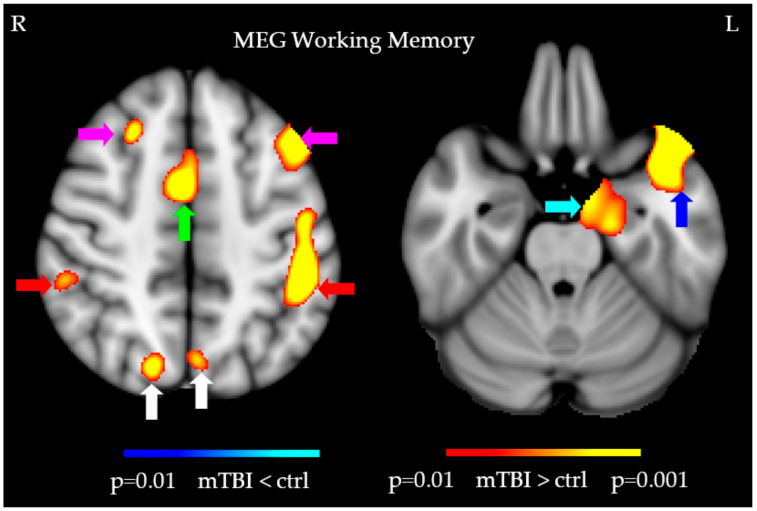
Evoked MEG working memory N-back task showed stronger (POST- vs. PRE-treatment) responses in individuals with mTBI who finished the *PATH* treatment than those who went through the *ReCollect* treatment. Stronger responses were from: bilateral dlPFC (magenta arrows); ACC (green arrows), PCC (red arrows), superior occipital gyri (white arrows), left anterior temporal lobe (blue arrow), and left hippocampus (cyan arrow).

Figure 10 shows the results of evoked MEG Working Memory (WM) N-back task, with stronger (POST- vs. PRE-treatment) responses in individuals with mTBI who finished the *PATH* treatment than those who went through the conventional *ReCollect* treatment. Stronger responses were from: bilateral dlPFC (magenta arrows); ACC (green arrows), PCC (red arrows), superior occipital gyri (white arrows), left anterior temporal lobe (blue arrow), and left hippocampus (cyan arrow). Two new cortical areas, the hippocampus and the anterior temporal lobe, improved in function, cortical areas not found previously after PATH neurotraining. The other cortical areas in the vision (superior occipital gyri), attention (ACC and PCC), and executive control (dlPFC) networks had stronger POST- vs. PRE-treatment MEG responses previously after PATH neurotraining [15,31].

## 4. Discussion

The present study investigated the efficacy of the *PATH* training program in mitigating cognitive deficits following mild traumatic brain injury (mTBI). Participants were randomized into three intervention groups: *PATH, ReCollect*, or Orientation Discrimination (OD). Cognitive performance was assessed across four key domains: Visual Working Memory (VWM), Auditory Working Memory (AWM), Processing Speed Index (PSI), and Selective Attention. Results demonstrated that *PATH* training yielded the most substantial improvements across all domains compared to the other interventions. Specifically, VWM improved by 35% in the *PATH* group, significantly exceeding gains observed in the *ReCollect* (15%) and OD (5%) groups. Similarly, AWM enhancements, though more modest, were significantly greater in the *PATH* group relative to OD, suggesting a broader transfer of benefits to auditory working memory processes. Moreover, the *PATH* group exhibited marked improvements in PSI, particularly within the Digit Symbol Coding subtest, outperforming the OD group. Lastly, significant advancements in Selective Attention, as measured by the Stroop test, were noted in the *PATH* group, characterized by faster response times and fewer errors relative to the other intervention groups. Collectively, these findings highlight the potential of *PATH* training to recalibrate fundamental sensory timing deficits and enhance higher-order cognitive functions.

These results align with existing research indicating that damage to magnocellular pathways within the dorsal visual stream contributes to chronic visual timing and attentional impairments following mTBI [72,73]. Prior studies have established that disruptions in dorsal stream functionality can result in persistent deficits in VWM and processing speed, presumably due to impaired sensory integration [74]. Supporting this perspective, the current study found that *PATH*, which directly targets low-level motion discrimination, facilitated significant improvements in both VWM and PSI. This suggests that recalibrating dorsal stream timing provides the necessary neural foundation for higher-order cognitive processes.

The robust gains in processing speed observed in *PATH* participants corroborate emerging evidence that rehabilitative programs aimed at optimizing dorsal stream function enhance visual discrimination speed and dynamic stimulus integration [74]. In contrast, the OD intervention, which targets the ventral stream, did not yield notable improvements in VWM, PSI, or Selective Attention, despite its established benefits for object perception and form recognition [75]. This discrepancy underscores the limited generalizability of ventral stream training to cognitive domains that rely on precise spatiotemporal processing.

Improvements in AWM within the *PATH* group are consistent with prior neuroimaging findings suggesting that interventions addressing foundational sensory timing deficits facilitate enhanced connectivity between posterior sensory and anterior executive control regions [76]. However, the smaller effect size for AWM relative to VWM and PSI indicates that the primary impact of *PATH* training may be most pronounced for visually mediated tasks. This likely reflects the greater reliance of VWM and PSI on dorsal stream reorganization, whereas AWM enhancements may depend on more diffuse, multimodal neural integration.

Enhancements in Selective Attention in the *PATH* group align with research demonstrating that recalibrating sensory timing can reduce the cognitive effort required to process motion-related cues, thereby freeing attentional resources for higher-order tasks [77]. The negligible improvements observed in the OD group suggest that refining ventral stream-oriented pattern discrimination alone is insufficient for modulating attention and inhibitory control—cognitive faculties that necessitate precise spatiotemporal synchronization.

### 4.1. Neurophysiological Correlates of TBI

Traumatic brain injury (TBI) leads to significant cortical modifications that contribute to cognitive impairment, independent of the initial injury severity. These changes include alterations in cortical thickness, disruptions in functional connectivity, and neuroinflammatory responses that can persist long after the acute phase of injury. TBI patients exhibit reduced cortical thickness in regions associated with executive function and working memory, particularly in the prefrontal cortex, as shown by studies utilizing neuroimaging techniques, such as magnetic resonance imaging (MRI) and functional MRI (fMRI) [78]. Moreover, resting-state fMRI studies have demonstrated widespread disruptions in functional connectivity within the default mode network (DMN), which correlate with cognitive deficits observed in TBI patients [79]. Chronic neuroinflammation, characterized by persistent microglial activation and elevated pro-inflammatory cytokines, has also been implicated in the long-term cognitive decline seen in TBI survivors [80]. These findings suggest that cognitive impairment following TBI is not solely attributable to the mechanical forces of injury but also to progressive cortical modifications that may exacerbate deficits over time. Future research should focus on identifying biomarkers of these cortical alterations to develop targeted interventions aimed at mitigating cognitive dysfunction in TBI patients.

### 4.2. Variability in Treatment Response

Individual differences in treatment response, as observed in Figure 4, Figure 5, Figure 6 and Figure 7, may stem from a combination of baseline impairment severity, adherence to training protocols, and neurophysiological variability among participants. Prior research suggests that pre-existing cognitive deficits significantly influence responsiveness to cognitive interventions, with those exhibiting more severe impairments at baseline often demonstrating greater gains due to a larger margin for improvement [81]. Additionally, participant engagement and adherence to intervention protocols have been shown to modulate outcomes, as higher session attendance and task compliance correlate with more robust cognitive improvements [82].

Neurophysiological factors, including individual differences in cortical plasticity and compensatory neural mechanisms, may also contribute to variability in cognitive recovery. Functional neuroimaging studies indicate that patients with greater baseline functional connectivity in dorsal stream networks exhibit enhanced learning capacity and cognitive recovery following TBI [83]. Moreover, variations in genetic and neurochemical profiles, such as differences in brain-derived neurotrophic factor (BDNF) expression, have been linked to differential neuroplastic responses and cognitive rehabilitation outcomes [84].

Finally, psychosocial factors, including motivation, stress levels, and mental health status, may mediate intervention efficacy. Studies suggest that individuals with higher intrinsic motivation and lower anxiety levels tend to engage more effectively with cognitive training, leading to greater improvements in executive function and working memory [85]. Given these complexities, future research should aim to identify specific predictors of treatment response, allowing for more tailored intervention strategies to optimize cognitive rehabilitation in individuals with mTBI.

### 4.3. Neurophysiological Correlates of Cognitive Gains

MEG neuroimaging results provide converging physiological support for the behavioral improvements following *PATH* training. Increased activation in the bilateral dorsolateral prefrontal cortex (dlPFC), anterior cingulate cortex (ACC), and posterior cingulate cortex (PCC) underscores the intervention’s effect on executive function and attentional control. These areas play a central role in working memory and cognitive flexibility, suggesting that *PATH* training strengthens core neural substrates responsible for task coordination and response inhibition.

Additionally, the engagement of the superior occipital gyri highlights the role of dorsal stream recalibration in improving processing speed and selective attention. Since the dorsal stream facilitates motion processing and visuospatial integration, enhanced activity in this region supports the observed behavioral improvements in PSI and attentional performance. Moreover, activations in the left anterior temporal lobe and hippocampus suggest that *PATH* training extends its impact to memory consolidation and retrieval (*Retraining Dorsal Visual Pathways Improves Cognitive Skills*, *Patent Pending*), likely contributing to the AWM gains. These neurophysiological findings affirm the potential of *PATH* training as a novel intervention that not only enhances cognitive function but also facilitates structural and functional cortical reorganization.

### 4.4. Quality of Life Improvements

The preliminary findings of the *PATH* neurotraining program also suggest promising enhancements in participants’ quality of life, as measured by the Rivermead Post-Concussion Symptoms Questionnaire (RPQ) and the SF-36 Role Limitations due to Emotional Problems (RE) subscale. Across all groups (*PATH*, *ReCollect*, and OD), significant improvements were observed in RE scores from PRE-test to POST-test, highlighting a meaningful reduction in perceived emotional role limitations over time. This trend was mirrored in RPQ outcomes, which indicated substantial symptom reduction following training. Notably, while no significant group differences or interactions were detected, the overall improvements emphasize the efficacy of the intervention in enhancing quality-of-life indicators regardless of group assignment.

The *PATH* neurotraining program appears particularly promising as a novel approach for addressing emotional and functional outcomes in mTBI populations. Participants in the *PATH* group demonstrated the largest gains in RE scores, suggesting that this intervention may hold unique potential for alleviating emotional role limitations. While these results remain preliminary, they warrant further investigation and refinement of the *PATH* methodology to optimize its impact on quality-of-life outcomes. Future research with larger sample sizes and more controlled experimental designs will be essential to fully elucidate the distinct benefits of *PATH* training.

### 4.5. Limitations

Several methodological and practical constraints must be considered in interpreting these results:

1. Sample Size and Generalizability: The relatively small number of participants limits the statistical power and may constrain the generalizability of findings. Although significant group differences emerged, replication with larger cohorts is warranted to bolster confidence in the robustness of these effects;
2. Outcome Measures: The standardized tests used in this study capture key cognitive domains but may not fully reflect participants’ everyday functional capacities. Future investigations should include ecologically valid tasks or instruments that measure real-world outcomes such as workplace performance and daily living skills;
3. Scope of Control Interventions: Although both *ReCollect* and OD are widely recognized rehabilitation approaches for mTBI, these interventions may not represent the full spectrum of existing cognitive rehabilitation paradigms. Consequently, the relative efficacy of *PATH* in comparison to other techniques remains to be fully elucidated.

### 4.6. Future Directions

Building on these promising results, forthcoming research should pursue several avenues:

1. Randomized Controlled Trials with Larger Samples: Multi-site trials with expanded sample sizes will help validate the efficacy of *PATH* training and clarify its applicability across diverse mTBI populations, including various etiologies and different timepoints post-injury;
2. Longitudinal Assessment of Durability: Although the current study demonstrated immediate post-training gains, it remains unclear whether these enhancements endure over extended periods. Future work should examine whether booster sessions or periodic refresher exercises help maintain or even augment the cognitive benefits of *PATH* training;
3. Functional and Quality-of-Life Outcomes: Future studies should include additional outcomes pertinent to real-world performance—such as success in academic or occupational settings, reductions in fatigue, and overall quality of life—to determine the broader relevance and translational impact of *PATH* training;
4. Hybrid or Sequential Training Approaches: Investigating whether combining *PATH* with established cognitive rehabilitation programs, such as conventional WM or executive function training, yields synergistic or additive improvements could help customize intervention protocols for specific cognitive profiles in mTBI populations.

## 5. Conclusions

This study provides compelling evidence for the efficacy of *PATH* training in ameliorating cognitive deficits associated with mTBI. Behavioral improvements in VWM, AWM, PSI, and Selective Attention were supported by MEG-based neurophysiological findings, which revealed enhanced activity in key cognitive and sensory processing regions. Increased functional activation in the prefrontal, cingulate, occipital, and temporal regions following *PATH* training further supports its role as a transformative neurorehabilitation approach.

Moreover, these neuroimaging results confirm that *PATH* training induces measurable changes in cortical activity, reinforcing its potential for facilitating lasting neural reorganization. The observed enhancements in quality-of-life measures further suggest that *PATH* training extends beyond cognitive performance to improve emotional and functional well-being.

While these findings are promising, future large-scale, multimodal neuroimaging studies and real-world functional assessments will be essential in determining the full scope and sustainability of *PATH* training’s impact. As research continues to refine and expand upon these findings, *PATH* neurotraining holds substantial promise as a viable, evidence-based intervention for mTBI rehabilitation.

## Data Availability

In the REDCap database at the University of South Alabama, and clinicaltrials.gov, Clinical Trials ID: NCT03655782, and in FITBIR database.

## Supplementary Materials

The following supporting information can be downloaded at: https://www.mdpi.com/article/10.3390/jcm14072273/s1, Figure S1: *ReCollect* Game SUMMARY for N-back task: Control your astronaut to touch the gem that matches the color of the gem the correct number back. For example, for the 2-Back level you selected a gem only if it was the same color as the one two gems back. On a 3-Back level, you selected the gems only if it was the same color as the one three gems back. On a 4-Back level, you selected the gems only if it was the same color as the one four gems back—Avoiding all other gems or your meter regressed.
