# Supplementary material for: Retraining Dorsal Visual Pathways Improves Cognitive Skills After a Mild Traumatic Brain Injury
Source: J Clin Med. 2025 Mar 26;14(7):2273. doi: 10.3390/jcm14072273 (PMC11989825; doi:10.3390/jcm14072273)
Supplement: Supplementary file 1 [file jcm-14-02273-s001.zip › jcm-3497436-supplementary.pdf]

Supplementary Materials

Human Subjects Characteristics

After the injury, a mTBI patient commonly experiences: headaches, nausea, vomiting, blurred or double vision, seeing stars or lights, balance problems, dizziness, sensitivity to light or noise, Tinnitus, drowsiness, fatigue or lethargy, irritability, depression, anxiety, sleeping more than usual, difficulty falling asleep, feeling "slowed down", "in a fog", or "dazed", difficulty concentrating, and difficulty remembering for at least 3 months [4]. A mTBI can result in substantial functional disability interfering with a patient's ability to return to work or school and can result in low levels of satisfaction with quality of life [16].

A patient with a mTBI who has had a traumatically induced physiologic disruption of brain function was referred by a doctor who made a diagnosis of mTBI, which included one or more of the following [16,71]:

1) any loss of consciousness from 5- 30 min (not longer than 30 min),
2) any loss of memory for events immediately before or after the accident for as much as 24 hours,
3) any alteration of mental state at the time of the accident (e.g. feeling dazed, disoriented, or confused),
5) Post-traumatic amnesia less than 24 hours,
6) a score of 19-25 on the Montreal Cognitive Assessment (MoCA) screening test, and
7) focal neurological deficits that might/might not be transient,
8) one or multiple concussions.

This definition includes [16,45]:

1. the head being struck,
2. the head striking an object, and
3. the brain undergoing an acceleration/deceleration movement (i.e, whiplash) without direct external trauma to the head.
4) symptomatology that, when linked to a traumatic head injury, can suggest the existence of a mild traumatic brain injury.

For this study, the following criteria were utilized:

Inclusion Criteria:

1) Diagnosis of mTBI by scoring between 18-28 on the Montreal Cognitive Assessment screening test and has working memory deficits,
2) between the ages of 18 to 60 years, when development and aging are not factors,
3) agrees to complete the study after hearing the time commitment involved,
4) has corrected 20/20 visual acuity, so can do PATH training (must see direction dim gray stripes move),
5) reads English fluently, so can follow instructions, and
6) can complete the PATH neurotraining task, by pushing the left or right arrow key on the computer.

Exclusion Criteria:

1) mTBI occurred less than 3 months ago to minimize the confound from the spontaneous recovery by only recruiting chronic mTBI subjects, and not recruiting mTBI subjects with mTBI less than 3 months, with no maximum time if still experiencing WM deficits.
2) post-traumatic amnesia longer than 24 hours,
3) diagnosis of epilepsy or seizure disorder in last 12 months,
4) diagnosis of major depressive disorder or severe anxiety,
5) answers 'Yes' to any of the questions on the Columbia Suicide Severity Rating Scale,
6) had a stroke or metabolic derangements causing cognitive impairments, i.e. alcohol or substance abuse,

And for those chosen to undergo MEG exams:

7) has extensive metal dental hardware (e.g., braces and large metal dentures; fillings are acceptable) or other metal objects in head, neck, or face areas that cause artifacts in MEG data, and are not removable during pre-processing,
8) has claustrophobia since MRI scanner is in small enclosed space, or
9) is pregnant.

Behavioral Measures to Assess Improvements in Cognitive Skills (Pre-Post tests).

1. **Visual Working Memory** (VWM) using *TIPS* (available from WPS: Western Psychological Services, Santa Monica, CA), having two distractor tasks to measure Sequential Processing: the subject had to remember a sequence of letters, that were shown one at a time for 2 seconds each, for sequences of from 2 up to 9 letters right after seeing the entire sequence of letters. Short Term WM was assessed by recalling the correct sequence of letters after counting from 1 to 10 numbers in sequence, starting at different initial numbers, slowly, and after repeating a short sentence with an animal subject for VWM. **Delayed Recall** was assessed by remembering all animal names in repeated sentences 3 minutes after finished the VWM tests. The *TIPS* took about 10 minutes, and is the most rapid standardized test to assess VWM.
2. **Auditory Working Memory (AWM)** using the *WAIS-4* Working Memory Index which consisted of two subtests: 1) the Digit Span subtest, where the subject had to repeat a list of spoken numbers, requiring the subject to remember subsequently more numbers: in the correct order, backwards, and in numerical sequence on three different subtests, and 2) the Letter-Number Sequencing subtest which required sequencing subsequently more numbers and letters in the correct numerical and alphabetic sequence. Presentation of the numbers and letters were timed for one second each for these working memory tests. This test took about 8 minutes to complete.
3. **Processing Speed** using *WAIS-4* Processing Speed Index (**PSI**): required two subtests: 1) the WAIS Symbol Search subtest which required subjects to scan a target group (two symbols) and search a group of 5 symbols, indicating whether one of the target symbols appeared in the search group, and 2) WAIS Digit Symbol Coding subtest, where the subject filled in boxes below digits with symbols that are paired with them in a key at the top of the page. Both of these subtests were timed for two minutes each. The scaled scores from each subtest were combined to create an overall Processing Speed Index (PSI) score, that was converted to a standardized percentile. These tests took about 6 minutes.
4. **Selective Attention** using the *DKEFS* Color-Word Interference test measuring time to say color of the printed word that denotes a different color (Stroop test). This test took about 8 minutes to complete.

These tests were chosen based on our pilot data assessments [15] and best practice (gold standard tests).

Strategies to Keep Subjects Focused on Intervention Task

Intervention training was implemented in a *high-fidelity* manner using: 1) training videos, 2) a detailed written protocol that all Research Assistants (RAs) were trained to follow, 3) all RAs were trained extensively to administer the neuropsychological tests and complete Movement Discrimination (*PATH)*, *ReCollect*, and Orientation Discrimination (OD) interventions before administering this training to subjects, and 4) being blind to which mTBI subjects are in which group when administering neuropsychological tests. The RAs took one quarter of independent research to learn how to administer the neuropsychological tasks and do the intervention training by making sure the subject was focused on the task. Each subject was trained initially one-on-one to ensure task was done correctly. All subjects completed supervised training to ensure training was done consistently, at same time of day, for 30 min 3 times/week for 12 weeks, and there were no distractions. Occasionally, the *ReCollect* task was done by a subject on their own, who then sent screen shots of their results. Text reminders were sent to each subject to remind them to complete the cognitive training the following day. If a subject experienced some discomfort, e.g. boredom, a slight headache, tiredness, or dizziness from visual or memory tasks, they were told to look away from screen, and take a short 1-2 minute break. These side effects were rarely experienced.

The three intervention training tasks were very simple to administer. Initially, the subject watched a 4-5 minute video on how to complete intervention training task. Since the task was so simple, no further instructions were usually needed. Each time the subject arrived, either in person or on zoom, they were seated at a MacBook Pro computer to do PATH or OD or Android tablet to do ReCollect task. Then their ID information was entered by the RA or subject to access subject's individual and summary data. The ReCollect training task took 30 minutes to complete. Both PATH and OD intervention tasks took 20 minutes to complete one complexity level, the program ending automatically when 20 patterns were completed, but could be ended before the session was completed by pushing the quit button (after 20 minutes of training). Both the PATH and OD training were followed by 10 minutes of WM exercises (Digit Memory Task) so the entire session took 30 minutes.

Since there were a total of 32 different complexity levels for both PATH and OD training, the intervention training took 12 weeks when one complexity level was completed 3 times each week, for a total of 18 hours. Sometimes more sessions were able to be completed in 20 minutes. When 16 levels were completed, the subject would restart PATH training at a lower complexity level, getting more experience discriminating 10-13.3 Hz movement. The main

supervision needed was to ensure the subject kept their eyes on the screen when pushing the left or right arrow key to signal the direction the center pattern moved or was tilted relative to the background pattern. As soon as the subject pushed the left or right arrow key, the next pattern appeared on the screen. If the subject was not paying attention, it was easy for the RA to tell, since they got it wrong (hear a short beep) when it should have been easy to get it right. Only if not paying attention or needed more practice would the contrast of the center stripes in PATH not be dim, or in the OD training, the pattern would be tilted noticeably from vertical. Each RA was taught how to be encouraging and help the subject learn the task quickly. If the subject was not paying attention, then the RA pushed the space bar for the subject to see another pattern, or 'x' key to restart this pattern so the subject had another chance to see movement dimly or determine the orientation more consistently. The RA was trained to be encouraging and shield the subject from distractions. The RA also looked at the computer data which lists the contrast or orientation discrimination threshold for each pattern to see if the subject needed more help next time which was entered into their notes. A bound notebook was kept for each subject, keeping detailed notes for each training session, including sleep quality, start and end times.

Visual Timing Intervention (PATH): Motion Direction-Discrimination Training

Participants received instructions on how to do the PATH movement-discrimination training by watching a 4-5 minute movie on PATH web-app dashboard. The subject sat 57 cm, an arm's length, in front of a computer monitor, with a display similar to the ones in Figure 2. During the presentation, the bars in the "fish-shaped" window in the center of the screen formed by a sinusoidal grating, moved left or right very briefly (<= 450 ms). When the screen went blank, the subject signaled the direction that the center pattern moved by pushing the left or right arrow key, see Figure 2. A brief tone was presented after incorrect responses, as well as the contrast of center pattern increasing one step. The program adaptively changed the contrast of the test pattern in order to keep the subject at 79% correct.

At the start of a session, both the test and background gratings were set to 5% contrast to ensure that the contrast of the test pattern was in the middle of the magnocellular contrast range [52]. The contrast and mean luminance, which was approximately 120 cd/m2, were measured using a Pritchard 1980A Spectra photometer and the Calibrate tab on the PATH web-app for administrator, presenting desired pattern of varying contrast from 0% up to 30% contrast, one step at a time. Each time the subject correctly identified the direction the fish stripes moved, the contrast of the test grating was lowered one step until the subject answered incorrectly. Following the first incorrect response, a double-staircase procedure, requiring subject to signal direction of movement correctly three times in a row before lowering contrast of test pattern [50], was used to measure the movement-discrimination contrast threshold. Lowering a subject's contrast threshold is what increases a subject's sensitivity to movement. This staircase procedure estimated the contrast threshold by using the most sensitive, repeatable measurements of contrast sensitivity possible [51]. A full training cycle of the movement-discrimination task required 20 contrast threshold determinations: for each of the four test spatial frequencies (0.25, 0.5, 1, and 2 cyc/deg) paired with each of the five background spatial frequencies (equal to test frequency or ±1 or ± 2 octaves from the test frequency), patterns chosen to optimally active magno-cells, see Figure 2A.

There were also levels of difficulty introduced by making the background pattern more similar to that in the fish, see center pattern in Figure 2A, by increasing the pattern's complexity level (Figure 2B), and by increasing the number of directions of movement from one to two directions of motion. The complexity level increased: 1) the number of sine wave components in the background from one (Figure 2A) to three harmonically related frequencies having a difference frequency equal to the test frequency (Figure 2B), shown previously to facilitate movement discrimination [42,43] by providing a wider background frame of reference, 2) the background contrast from 5% to 10% to 20%, to increase the amount of parvocellular activity, since magno-cells saturate at 10% contrast [52], and 3) the pattern's speed of movement after every 4 complexity levels, increasing from 6.7 to 8 Hz (in theta range) to 10 to 13.3 Hz (in alpha range), so that the subject was challenged as the training progressed. Faster speeds of movement, 10 Hz to 13.3 Hz, were too fast to be trained on until slower speeds of movement had been trained.

Control Intervention: Orientation Discrimination Task

The control intervention was Orientation Discrimination (OD) training that was identical to PATH training except instead of low contrast sine wave gratings moving left or right, high contrast (97%) stationary test and background sine wave gratings were used, both black and red and black and white gratings, see patterns in Figure 3. The color of

the center pattern was either: 1) white and black surrounded by a vertical red and black grating for all odd complexity levels, or 2) red and black surrounded by a vertical white and black grating for all even complexity levels, see Figure 3. The vertical background grating served as a frame of reference to judge whether the center pattern was tilted left or right. The complexity level increased so the subject was continually challenged. Each level of complexity consisted of 20 different test-background spatial frequency combinations. The complexity level started with lower spatial frequencies, systematically progressing to higher spatial frequencies for 16 levels of complexity. For each of four test spatial frequencies. progressing from (1.5, 2, 3, and 4 cyc/deg) to (2, 3, 4, 5 cyc/deg) to (3, 4, 5, 6) to (4, 5, 6, 7 cyc/deg), as complexity level increased, each paired with five background spatial frequencies (equal to test frequency or ±1 or ± 2 octaves from the test frequency), patterns chosen to optimally active parvo-cells. There were 80 different combinations of center and background spatial frequencies for 16 levels of complexity. These spatial frequency combinations were similar to those used in PATH training except that they were shifted up so were about four times higher in spatial frequency. For the OD task, the subject pushed the left arrow key when the test pattern was tilted left and the right arrow key when pattern was tilted right. Otherwise, the two training tasks used the same paradigm.

ReCollect Task Intervention

Here we used *ReCollect the Study* (a cross-platform WM game developed by Dr. Aaron Seitz, Northeastern University). In this task, participants were required to compare each item to the item that they saw n-items back in the sequence. For example, in Figure S1 below, the second yellow item is a target, as it matches the item 2-back in the sequence. The participant played a simple game where they controlled the movement of an astronaut that needed to collect correct gems, avoid incorrect gems, and also obstacles, to succeed at the game. If the participants performed well on a 1-back task, then they were advanced to the 2-back, 3-back, 4-back, etc where they made similar matches but to earlier items in the sequence. This gamified task consisted of a color n-back with 6 colors (a new color every 3 seconds, requiring subjects respond to targets by tapping the screen and navigating the astronaut to the targets, and avoiding the distractors) with 30% targets, and where the n-level changed every 2 minutes depending on performance (increased if performance was >85% and decreased if <75%). Sessions consisted of 10 ~2 minute blocks, each with n-level as determined by the adaptive procedure and with user paced breaks between blocks.

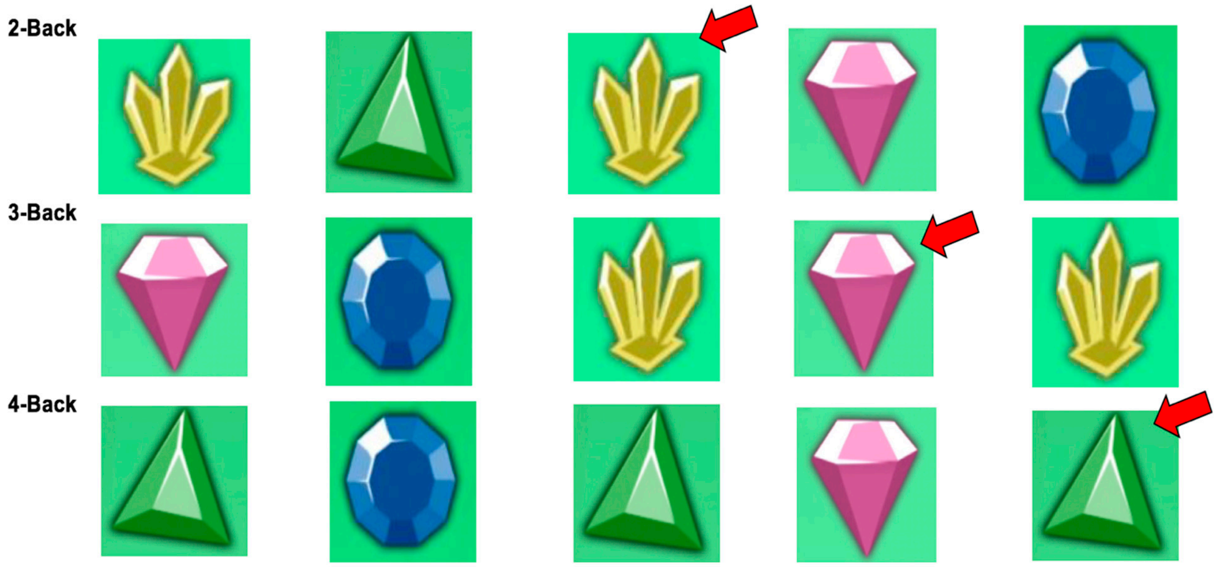

Figure S1. *ReCollect* Game SUMMARY for N-back task: Control your astronaut to touch the gem that matches the color of the gem the correct number back. For example, for the **2-Back** level you selected a gem only if it was the same color as the one **two gems back**. On a **3-Back** level, you selected the gems only if it was the same color as the one **three gems back.** On a **4-Back** level, you selected the gems only if it was the same color as the one **four gems back** – Avoiding all other gems or your meter regressed.
